# Research Progress on Preparation, Mechanism, and Clinical Application of Nanofat

**DOI:** 10.1093/jbcr/irab250

**Published:** 2022-01-07

**Authors:** Pengbing Ding, Enhang Lu, Guan Li, Yidan Sun, Wenhui Yang, Zhenmin Zhao

**Affiliations:** Department of Plastic Surgery, Peking University Third Hospital, Beijing, China; Department of Plastic Surgery, Peking University Third Hospital, Beijing, China; Department of Plastic Surgery, Peking University Third Hospital, Beijing, China; Department of Plastic Surgery, Peking University Third Hospital, Beijing, China; Department of Plastic Surgery, Peking University Third Hospital, Beijing, China; Department of Plastic Surgery, Peking University Third Hospital, Beijing, China

## Abstract

Autologous adipose tissue is an ideal soft tissue filling material in theory, which has the advantages of easy access, comprehensive source, and high biocompatibility and is now widely used in clinical practice. Based on the above benefits of autologous fat, autologous fat grafting is an essential technique in plastic surgery. Conventional macrofat is used to improve structural changes after soft tissue damage or loss caused by various causes such as disease, trauma, or aging. Due to the large diameter of particles and to avoid serious complications such as fat embolism, blunt needles with larger diameters (2 mm) are required, making the macrofat grafting difficult to the deep dermis and subdermis. Nanofat grafting is a relatively new technology that has gained popularity in cosmetic surgery in recent years. Nanofat is produced by mechanical shuffling and filtration of microfat, which is harvested by liposuction. The harvesting and processing of nanofat are cost-effective as it does not require additional equipment or culture time. Unlike microfat, nanofat particles are too small to provide a notable volumizing effect. Studies have shown that nanofat contains abundant stromal vascular fraction cells and adipose-derived stem cells, which help reconstruct dermal support structures, such as collagen, and regenerate healthier, younger-looking skin. Moreover, the fluid consistency of nanofat allows application in tissue regeneration, such as scars, chronic wounds, and facial rejuvenation. This article reviews the current research progress on the preparation, mechanism, and clinical application of nanofat.

Fat grafting has the advantages of wide source, convenient acquisition, good biocompatibility, and less surgical trauma, and was first applied in soft tissue filling, such as defect repair and deformity correction. In recent years, with the deepening of research, it has been found that stromal vascular fraction (SVF) cells and adipose-derived stem cells (ADSCs) in transplanted fat have multilineage differentiation ability, can differentiate into adipocytes, osteocytes, chondrocytes, and nerve cells in different environments, and can secrete cytokines such as vascular endothelial growth factor (VEGF), hematopoietic growth factor, and basic fibroblast growth factor (bFGF), which can effectively improve the survival rate of transplanted fat.^1^ There are three types of fat used for fat grafting: macrofat, microfat, and nanofat^2^: 1) Macrofat: fat grafts are harvested with fat grafts larger than 2.4 mm, is indicated for filling a wide range of sites such as the breast and buttocks and is generally injected using blunt needles with a diameter of 2 mm and above.^3,4^ 2) Microfat: fat grafts are harvested using a cannula with a hole diameter ranging from 1.2 to 2.4 mm, an emulsifier with a hole diameter of 1.2 mm, and a parcel diameter of less than 1.2 mm. Using the mircofat on the forehead, eyelids, brows, as well as the nose and hands, is highly advised, without concern for possible complications when injecting larger fat particles through bigger instruments.^5,6^ 3) Nanofat: fat grafts extracted using a 1.2 to 2.4 mm diameter cannula, 400 to 600 µm emulsifier, and 400 to 600 µm parcel diameter. The intradermal use of nanofat for the treatment of superficial rhytids is highly recommended. Macrofat grafting is mainly used to establish large volumes. Keeping as many viable adipocytes as possible is important, especially in cases of breast reconstruction.^7^ Due to the inability to establish large fat volumes, nanofats are not suitable for these indications. Microfat and nanofat injections are typically performed concurrently.^2^ Microfat offers soft tissue structural support and filling effect, whereas nanofat enhances skin quality and promotes tissue regeneration in applications of scars, chronic wounds, and facial rejuvenation.^8,9^

The concept of nanofat was first proposed by Tonnard et al in 2013,^7^ where the obtained particulate fat was extracted for mechanical emulsification followed by filtration to obtain SVF-gel rich in ADSCs. By repeating or improving Tonnard’s nanofat preparation method, researchers have successively reported the preparation techniques of nanofat.^10–14^ Studies indicated that all mature adipocytes in nanofat were destroyed.^15,16^ However, there were still many mesenchymal stem cells (MSCs) with great stem cell proliferation ability, which also had a number of differentiation functions and paracrine abilities and were the main components in nanofat. Nanofat is rich in SVF and ADSCs, which can regulate neovascularization and tissue regeneration through paracrine effects or directly differentiate into adipocytes to improve fat graft survival rates and play an essential role in tissue repair and regeneration.^17^

## MATERIALS AND METHODS

### Harvesting and Preparation of Nanofat

#### Tonnard Technique

To obtain nanofat, a patient’s fat tissue is first harvested from a donor area, such as the hips, abdomen, or back.^7^ Negative pressure liposuction is performed using a standard liposuction device. The liposuction tube was selected as a 3-mm diameter cannula with multiple orifices, a sharp side hole of 1 mm in diameter. The obtained fat is rinsed with normal saline and filtered through a sterile nylon cloth with a 0.5-mm pore size. The filtered fat is transferred into two 10-mm syringes and connected with Luer-Lok connecting tube. After pushing back and forth about 30 times, the granular fat is seen to become chylous. Finally, the emulsified fat is filtered once again on the nylon gauze with a diameter of 0.6 mm. The collected nanofat is injected intradermally with a 27-G needle.

#### Other Techniques

Based on the Tonnard technique, different scholars have modified it accordingly.

Liang et al and Wei et al transferred the fat between two 20-cc syringes connected by a medical female-to-female Luer lock connector to achieve mechanical emulsification.^10,11^ Bi et al suggested that nanofat prepared by Tonnard were obtained by physical methods, which severely disrupt the activity of adipocytes during the processing of fat.^12^ The authors reported a new approach to nanofat by chemical digestion with type I collagenase followed by centrifugation. The obtained nanofat includes adipocytes, adipose progenitor cells, and MSCs. The obtained nanofat was named “Vivo nanofat.” They determined that the Vivo nanofat contained active components of adipose tissue-derived MSCs by cell colony formation assay, flow cytometry, adipogenesis, and osteogenesis induction. The study also found that Vivo nanofat contained more stem cell active components than nanofat, which expressed intercellular markers and had multilineage differentiation potential. The experiments in mice showed that Vivo nanofat had a higher survival rate and a lower absorption rate.^10^ The size of the transplanted Vivo nanofat was 0.81 ± 0.07 cm^3^, which was more significant than that of nanofat 0.50 ± 0.17 cm^3^. Pallua et al proposed a two-step centrifugation protocol based on the Tonnard technique and named the obtained concentrated nanofat as lipoconcentrate.^13^ The study indicated that the lipoconcentrate contained a significantly higher amount of ADSCs and endothelial progenitor cells than nanofat centrifuged one time. Lo et al concluded that the Tonnard technique lost a large number of ADSCs during the preparation of nanofat and proposed the concept of second-generation nanofat (Nanofat 2.0),^14^ which have the same preparation process as the Tonnard technique, except that the last step, that is, the filtration of emulsified fats, is omitted. The results of the study suggested a higher ADSCs content and better differentiation ability in Nanofat 2.0.

### Mechanism of Nanofat

Nanofat is a natural emulsified suspension derived from adipose tissue. Nanofat can promote skin regeneration because of the presence of stem cells and growth factors.^7^ Studies have shown that grafted adipocytes in adipose tissue are destroyed after emulsification.^14,18^ Still, the emulsification process does not significantly affect several differentiation abilities such as stem cell yield, viability, or adipogenesis. Hence, stem cells, instead of grafted adipocytes, are more likely to be responsible for the regenerative effect on skin.

Although nanofat does not contain mature adipocytes, it is rich in a large number of SVF, which contains different types of cells such as endothelial cells, granulocytes, monocytes, and macrophages, and also includes a large number of MSCs.^15^ ADSCs are MSCs found within the SVF of subcutaneous adipose tissue. ADSCs self-renew display a multilineage developmental plasticity used in various tissue repair and regeneration clinical studies.^19^ Matsumoto et al have demonstrated that the addition of ADSCs to injectable autologous particulate fat improves the survival of transplanted fat.^20^

SVF also has regenerative properties compared with ADSCs, and it is easy to collect.^19,21^ Tonnard et al conducted a comparative analysis of nanofat, macrofat, and microfat for basic research.^7^ After isolation and culture of stem cells, it was found that there were (1.9–3.0) × 10^6^ SVF-derived active stem cells per 100 ml of nanofat, which was not dependent on the processing method of adipose tissue, and there were (0.1–0.2) × 10^6^ SVF-derived CD34(+) cells. The SVF and CD34(+) cells were cultured in a standard medium, in which adherent cells formed a monolayer and presented fibroblast morphology, which was not significantly different from macrofat and microfat. The SVF and CD34(+) cells in nanofat could differentiate into mature adipocytes, which were not qualitatively and quantitatively different from macrofat and microfat. The CD34(+) cells are proliferative and have a higher ability to form colonies.^22^ The CD34(+) adipose tissue resident macrophages possessed characteristics similar to ADSCs, which play an important in tissue maintenance and remolding.^23^ Also, MSCs have been shown to improve fat graft viability, mainly by enhancing angiogenesis.^24,25^

Although microfat contains adipocytes that are intact and alive, as well as their surrounding cell milieus such as SVF, ADSCs, and CD34(+), it requires needles larger than 27 G for smooth fat injection.^2,7^ However, nanofat with the above mentioned benefits can achieve skin regenerative purposes with smaller needles by reducing the number of adipocytes.

Yu et al transplanted nanofat combined with macrofat into the subcutaneous tissue in nude mice.^15^ The results at 12 weeks after transplantation showed that the combination group exhibited higher graft weight and volume retention, better tissue structure, and higher capillary density than the macrofat group. The study suggested that cotransplantation nanofat can strongly and effectively enhance neovascularization and fat graft survival. Moreover, nanofat contains dead adipocytes, releasing cytokines, and attracting macrophages to release growth factors, thereby stimulating the differentiation of ADSCs and tissue regeneration.^26^

ADSCs have wound repair function and multilineage differentiation ability, differentiated into adipocytes, osteoblasts, chondrocytes, and self-renewal ability under certain conditions.^27^ ADSCs have also been found to have paracrine functions and can secrete various growth factors such as VEGF and bFGF.^18,28^ VEGF and bFGF play a key role in fat transplantation.^29^ VEGF binds to vascular endothelial growth factor receptor 2 to promote vascular endothelial cell proliferation and cell migration, thereby inducing the regeneration of blood vessels, and increasing the permeability of blood vessels and the reconstruction of the neovascular network,^30^ hence improving the fat graft survival and reducing fat graft resorption.^31,32^ bFGF contributes to angiogenesis and regeneration and can also activate various repair cells.^33^ bFGF can act on endothelial cell chemokines and promote the division of endothelial cells, so it has a vital role in promoting adipocyte proliferation and differentiation and skin regeneration, and wound repair.^34,35^ ADSCs can secrete various antifibrotic factors, such as interleukin-10 and hematopoietic growth factor, through paracrine effects.^36^ These substances can participate in the repair process of mucocutaneous wounds, inhibit scar formation while improving skin texture, and play an excellent therapeutic effect in facial rejuvenation and scars.^37,38^

Like mircofat, adipose tissue extracellular matrix also exists in nanofat, which contains substances that can induce apoptosis of fibroblasts and inhibit the excessive secretion of fibrogenic substances, such as resistin and tumor necrosis factor.^39^ ADSCs have also been found to inhibit the expression of scar-promoting proliferation genes in hypertrophic scar fibroblasts (HSFs) through paracrine effects, such as type I and III collagen, transforming growth factor β1, interleukin-6, alpha-smooth muscle actin, fibronectin, and connective tissue growth factor, while promoting the expression of antifibrotic genes, such as decorin and matrix metalloproteinase-1.^40^

In addition to inhibiting scar hyperplasia by secreting antifibrotic factors, ADSCs in nanofat may have other mechanisms against fibrosis. Redd et al^41^ have shown that the inflammatory environment of the wound healing can stimulate MSCs to initiate immunomodulatory effects, such as upregulating the expression of prostaglandin E2 and cyclooxygenase-2, thereby inhibiting or alleviating local inflammatory response and immune dysfunction. Besides, ADSCs can also inhibit the protein expression levels of transforming growth factor β1 and its intracellular signaling pathway-related molecules such as phospho-mothers against decapentaplegic homolog 2 (p-sma2), p-smad3 in HSF, thereby inhibiting the proliferation, migration, and contraction of HSF, and ultimately achieving the inhibition of scar hyperplasia.^18,40^

## CLINICAL APPLICATION OF NANOFAT

### Application in Facial Skin Rejuvenation

Since nanofat has no filling ability, nanofat grafting achieves the purpose of facial skin rejuvenation by injecting regenerative cells and extracellular elements.^42^ According to the study of Tonnard,^7^ the results have demonstrated an improvement in skin quality immediately after nanofat grafting in 67 patients in the treatment of facial wrinkles and photoaged skin, reaching the best 4 to 6 months after surgery. Besides, 6 months after nanofat transfer, the clinical effect of facial skin rejuvenation in the surgical area is significant, without complications or other adverse reactions. Mesguich et al^43^ performed intradermal injection of nanofat with a small needle into perioral wrinkles in four elderly patients aged 50 to 59 years and found significant improvement in perioral wrinkles and high patient satisfaction after 4 months. Liang et al^8^ used nanofat combined with platelet-rich fibrin injection to treat facial skin aging, and the results showed that 103 patients who received nanofat combined with platelet-rich fibrin injection had more significant improvement in facial skin texture and satisfaction than 128 patients who received hyaluronic acid injection, without causing any complications (eg, infection, allergic reactions, or paresthesia). In vitro experiments have shown that ADSCs can whiten the skin by inhibiting melanin synthesis by downregulating the expression of tyrosinase and tyrosinase-associated protein 1.^44^ Oh et al^45^ applied nanofat to treat 19 cases of lower eyelid skin pigmentation, and follow-up of 2 to 4 months revealed that the dark coloration of all patients was significantly improved, with only mild ecchymosis and swelling after surgery.

### Application in Scar Repair

Because the scar tissue contains fibrotic tissue and has a hard texture, the injection of fat into the scar requires that the injection needle be thin enough. Nanofat particles have a small diameter, smoothly pass through a 27-G needle, and have a relatively large contact area with the scar after injection, so nanofat has unique advantages in treating scars repair. Bhooshan et al^46^ applied nanofat for local injection therapy in the scar, and the results demonstrated that nanofat scar injection could effectively improve scar characteristics and symptoms. Uyulmaz et al^9^ injected nanofat intradermally or directly into the scar tissue at the scar, wrinkle, or skin discoloration in 52 patients. The skin quality was assessed according to the scoring system, and patient satisfaction was recorded. Follow-up of 155 ± 49 days revealed that 40 cases of scars, 6 cases of wrinkles, and 6 cases of skin discoloration were effectively treated. The quality of scars was significantly improved after treatment high patient satisfaction. The results demonstrated that nanofat transplantation helped to improve scars, wrinkles, and skin discoloration. Tenna et al injected nanofat subcutaneously and found that nanofat treatment was effective in atrophic scars. Tenna et al^47^ treated acne scar patients aged 18 to 52 years with platelet-rich plasma plus nanofat for an average of two treatments, some of which were followed by fractional CO_2_ laser resurfacing treatment. The results showed that platelet-rich plasma plus nanofat with or without laser treatment had good clinical effects on acne-induced scars with increased skin thickness. Kemaloğlu^48^ found that the outcome was unsatisfactory after split-thickness skin graft transplantation for problematic wounds, and follow-up for 6 months after combination therapy of split-thickness skin and nanofat grafting revealed significant results and good flexibility at the skin graft site. Jan et al^49^ injected “unfiltered nanofat” into the subcutaneous or intradermal layers of 48 postburn facial scars and found significant improvements in both pigmentation and flexibility in scar tissue after 6 months of follow-up.

### Application in Other Aspects

In recent years, nanofat has been used in joint diseases. Mahmmood and Shihab^50^ applied nanofat injection on 11 patients (3 males and 8 females) diagnosed with temporomandibular joint disease, aged between 18 and 34 years, of which 3 cases undergo unilateral intra-articular nanofat injection, and 8 cases undergo intra-articular nanofat injection. The results showed that the degree of pain was reduced, and the maximum mouth opening was increased in patients, suggesting that the nanofat injection is effective, safe, and simple in the treatment of temporomandibular joint disease, which is acceptable to patients without significant adverse reactions. Nanofat has also been applied to vulva lichen sclerosus with good clinical results. Tamburino et al^51^ injected nanofat into the subcutaneous layer of the labia majora and clitoris. Although some patients had a recurrence of clitoral phimosis, the skin texture and elasticity were improved, and the postoperative itching and burning sensation were also reduced. Also, compared with lipofilling grafting, nanofat grafting is easier to apply to scalp areas requiring high-precision injections. This technique can be used as a supplement for hair follicle transplantation or as a regenerative treatment for alopecia.^52^ Although it has been confirmed that fat transplantation can promote the regeneration of shed eyebrows and improve alopecia caused by scleroderma,^53^ the effect of nanofat grafting on hair still needs to be confirmed by more studies.

## CONCLUSION AND PROSPECTS

The average diameter of nanofat is 400 to 600 µm, which can be easily injected with a 27-G needle or even a smaller diameter needle. The invention of nanofat provides a simple technique for preparing SVF and ADSCs and expands the therapeutic range of fat transplantation. It offers new ideas for improving the survival rate of granular fat transplantation, the treatment of scars, and the local injection of delicate parts of the body, especially around the eyes, mouth, hands, and neck.

However, there are still limitations in the use of nanofat for autologous fat transplantation: 1) during the preparation process, the optimal number of back and forth injections, the mixed proportion that can achieve the maximum fat survival rate, and the number of injections that can achieve significant clinical effects remain to be studied; 2) there is a lack of long-term clinical effect observation on the improvement of skin and scar quality by nanofat; 3) nanofat has a certain positive effect on promoting fat transplantation, but there is still a lack of a large number of clinical controlled studies, especially long-term clinical effects, and its specific mechanism of action remains to be proved by further studies.

It is believed that in the near future, nanofat as an option and supplement to fat grafts will provide a potentially clinically feasible method in plastic and reconstructive surgery.

## Notes

*Funding:* This project was supported by the National Natural Science Foundation of China (81571925) and the Key Clinical Projects of Peking University Third Hospital (No. BYSY2018061).

*Authors’ contribution:* Pengbing Ding was dedicated to the guarantor of integrity of the entire study, study concepts, study design, definition of intellectual content, intellectual content, experimental studies, data acquisition, data analysis, statistical analysis, manuscript editing, and manuscript review; Enhang Lu carried out the intellectual content, experimental studies, and manuscript review; Guan Li, Yidan Sun, and Wenhui Yang were involved in the clinical studies; Zhenmin Zhao performed the study concepts and manuscript review. All authors have read and approved this article.
